# Outcome reporting in neonates experiencing withdrawal following opioid exposure in pregnancy: a systematic review

**DOI:** 10.1186/s13063-020-4183-9

**Published:** 2020-03-12

**Authors:** Flora Shan, Sonya MacVicar, Karel Allegaert, Martin Offringa, Lauren M. Jansson, Sarah Simpson, Wendy Moulsdale, Lauren E. Kelly

**Affiliations:** 1 Department of Pediatric and Child Health, University of Manitoba, 405 Chown, 753 McDermot Ave., Winnipeg, MB R3E0T6 Canada; 2 School of Health and Social Care, Edinburgh Napier University, Edinburgh, UK; 3 Department of Development and Regeneration, and Department of Pharmaceutical and Pharmacological Sciences, KU Leuven, Leuven, Belgium; 4 Department of Clinical Pharmacy, Erasmus MC, Rotterdam, The Netherlands; 5 Department of Paediatrics, University of Toronto, Child Health Evaluative Services, The Hospital of Sick Children, Toronto, Canada; 6 Department of Pediatrics, Johns Hopkins University School of Medicine, Baltimore, MD USA; 7 Special Care Nursery, Women’s and Infants’ Program, St. Joseph’s Healthcare, Hamilton, Canada; 8 Dan Centre for Women and Babies, Neonatal Intensive Care Unit, Sunnybrook Health Sciences Centre, Toronto, Canada; 9 Clinical Trials Platform, the George and Fay Yee Centre for Healthcare innovation, Winnipeg, Canada

**Keywords:** Neonatal withdrawal syndrome, Neonatal abstinence syndrome, Neonatal opioid withdrawal syndrome, Core outcome set, Opioid exposed newborn baby, Maternal opioid use disorder

## Abstract

**Background:**

Neonatal withdrawal secondary to in utero opioid exposure is a growing global concern stressing the psychosocial well-being of affected families and scarce hospital resources. In the ongoing search for the most effective treatment, randomized controlled trials are indispensable. Consistent outcome selection and measurement across randomized controlled trials enables synthesis of results, fostering the translation of research into practice. Currently, there is no core outcome set to standardize outcome selection, definition and reporting. This study identifies the outcomes currently reported in the literature for neonates experiencing withdrawal following opioid exposure during pregnancy.

**Methods:**

A comprehensive literature search of MEDLINE, EMBASE and Cochrane Central was conducted to identify all primary research studies (randomized controlled trials, clinical trials, case-controlled studies, uncontrolled trials, observational cohort studies, clinical practice guidelines and case reports) reporting outcomes for interventions used to manage neonatal abstinence syndrome between July 2007 and July 2017. All “primary” and “secondary” neonatal outcomes were extracted by two independent reviewers and were assigned to one of OMERACT’s core areas of “pathophysiological manifestation”, “life impact”, “resource use”, “adverse events”, or “death”.

**Results:**

Forty-seven primary research articles reporting 107 “primary” and 127 “secondary” outcomes were included. The most frequently reported outcomes were “duration of pharmacotherapy” (68% of studies, *N* = 32), “duration of hospital stay” (66% of studies, *N* = 31) and “withdrawal symptoms” (51% of studies, *N* = 24). The discrepancy between the number of times an outcome was reported and the number of articles was secondary to the use of composite outcomes. Frequently reported outcomes had heterogeneous definitions or were not defined by the study and were measured at different times. Outcomes reported in the literature to date were mainly assigned to the core areas “pathophysiologic manifestations” or “resource use”. No articles reported included parent or former patient involvement in outcome selections.

**Conclusions:**

Inconsistent selection and definition of primary and secondary outcomes exists in the present literature of pharmacologic and nonpharmacologic interventions for managing opioid withdrawal in neonates. No studies involved parents in the process of outcome selection. These findings hinder evidence synthesis to generate clinically meaningful practice guidelines. The development of a specific core outcome set is imperative.

## Background

Neonatal abstinence syndrome (NAS) is a postnatal withdrawal syndrome that occurs after fetal exposure to substances (for example, opioids, antidepressants and stimulants) in utero [1]. Among neonates exposed to opioids in utero, 55–94% [2] will demonstrate the clinical manifestations of NAS which predominantly involve central nervous system irritability, autonomic dysregulation and gastrointestinal dysfunction [1]. Typically, opioid-exposed neonates are observed in hospital for 3 to 7 days for the development of NAS symptoms prior to discharge [2]. Approximately 50–80% of these infants exhibit moderate to severe withdrawal symptoms requiring pharmacologic management in addition to supportive nonpharmacologic interventions [3, 4]. The use of pharmacotherapy varies depending on whether the neonate meets the “treatment threshold” per the diagnostic criteria utilized (i.e., the Finnegan Scale, Lipsitz score, or the Eat Sleep Console model) which varies between centers. The average length of stay for a neonate experiencing NAS is 16–23 days, accounting for monitoring and stabilization of symptoms with pharmacotherapy [5].

In recent years, NAS has become a global concern with increasing prevalence rates ranging between 2.7 and 5.8 per 1000 live births [5–7]. In the USA between 2009 and 2012, the aggregate hospital charges for NAS increased from $732 million to $1.5 billion dollars [5]. A similar trend is documented in Canada, with tripling of the daily hospital beds occupied by neonates with NAS from 19.7 beds in 2003 to 69.4 beds in 2014 [8]. The actual burden of disease is likely inaccurate given the complexity of exposure, genetic factors that may increase the risk for withdrawal, patterns of recognition and reporting, along with extensive variability in regional substance misuse and diagnostic criteria. The extent to which different prevalence rates vary by hospital related to recognition or recording requires further investigation. Robust evidence is lacking on the long-term consequences (i.e., medical, neurodevelopmental or psychosocial) for the individual who has experienced NAS [3]. The current increase in NAS diagnosis is believed to be multifactorial and driven by improvements in screening, awareness and practice guidelines, and by the increasing rates of both illicit drug use and prescription of opioids/psychotropic medications during pregnancy [9, 10]. Historically, NAS presentation was primarily due to in utero opioid exposure [3]. Neonatal opioid withdrawal syndrome (NOWS) is a subset of NAS reflecting neonatal withdrawal following exposure to opioids in pregnancy. The later, more specific definition has recently been adopted and may not be reflected in previously published reports. As polysubstance exposures during pregnancy are becoming more prevalent, reportedly as high as 65% in one study [11] and even higher with the inclusion of alcohol and tobacco, NAS is becoming an increasingly complex syndrome with less predictable time of onset, severity and response to pharmacologic therapy [12, 13]. From the clinical perspective, there has been a paradigm shift in infant assessment and treatment initiation (using thresholds evaluated by subjective features of withdrawal to using the Eat, Sleep, Console method) [14] and the emergence of novel pharmacokinetic- and pharmacodynamic-based dosing protocols [15]. The ever-increasing burden of disease, evolving complexity in presentation and changing pharmacotherapy initiation models have led to a growing interest in NAS research from scientists, clinicians and policy makers.

In 2013, the World Health Organization evaluated all available evidence on identifying and managing neonates withdrawing from in utero substance exposure. The quality of evidence behind the recommendations was determined to be “very low” per the Grading of Recommendations, Assessment, Development and Evaluations framework [16, 17]. This paucity of evidence is reflected in the heterogeneity of clinical care for infants at risk for NAS. National studies in Canada [18], the USA [19], the UK and Ireland [20] demonstrated significant variability in NAS/NOWS assessment tools, types and doses of opioids utilized, and addition of adjuvant agents across neonatal intensive care units. Current economical assessments continue to show a substantial rise in NAS-related morbidity and costs [21–23]. With stakeholders examining the same topic through various lenses from bench-to-bedside to policy development, it is imperative to establish clinically relevant and standardized outcome measures. At present, there is no consensus on what to measure nor consistent units of measurement in research and quality improvement initiatives on neonates with NAS/NOWS.

The purpose of this study is to evaluate consistency in outcomes reported in all observational and interventional studies of neonates exposed to opioids during pregnancy who develop withdrawal (NAS/NOWS). We will assess neonatal outcomes reported in all studies investigating pharmacological and nonpharmacological interventions for infants that were exposed to opioids (such as methadone, buprenorphine, oxycodone, or prescription opioids with or without concomitant use of other illicit substances) in utero and who are diagnosed with NAS or NOWS in the postnatal period.

## Methods

### Protocol and registration

This initiative has been prospectively registered with Core Outcome Measures in Effectiveness Trials (COMET) [24]. The complete study protocol has been published [25] and is available at https://trialsjournal.biomedcentral.com/articles/10.1186/s13063-016-1666-9

### Eligibility criteria

Primary research studies including randomized controlled trials, clinical trials, case-controlled trials, uncontrolled trials, observation cohort studies, clinical practice guidelines, and case reports of any interventions used to manage NAS/NOWS were analyzed. For the purpose of this review, studies of pharmacological and nonpharmacological management of neonates exposed to opioids (including methadone, buprenorphine, oxycodone, prescription opioids) in utero who are diagnosed with NAS were included. We included all co-exposures based on the rationale that they commonly occur and that ICD 9 diagnostic coding does not separate withdrawals related only to opioids. All publications identified from the published search strategy between January 2007 and June 2017 were included. The rationale behind the time restriction is that a Cochrane review was published in 2009 encompassing studies reported prior to 2007 and a shift in clinical practice away from ubiquitous use of deodorized dilution of opium [26]. The steering committee felt that there was limited value in evaluating what outcome measures were used before widespread use of the Finnegan assessment tools and oral morphine treatment weaning.

### Search strategy

The search strategy (Additional file 1) was developed in conjunction with a reference librarian at the Hospital for Sick Children on Ovid MEDLINE 1946 to present with daily update, Ovid MEDLINE in-process and other nonindexed citations. The search was also applied to EMBASE and Cochrane Central on 6 June 2019. The latest Cochrane review was published in 2010. Reference lists of four recently published systematic reviews of NAS were evaluated. In addition to the electronic search strategy completed as above, ClinicalTrials.gov was reviewed and identified 37 studies for ongoing trials related to NAS. Bibliographies of all included studies and systematic reviews were reviewed to identify relevant articles not generated in the search. In addition, the steering committee reviewed the list of articles to ensure comprehensiveness and to provide related articles not identified by the search strategy.

### Study selection

Two independent reviewers (LEK and SM) screened titles and abstracts resulting from all the search strategies in EndNote X6. For studies that were deemed eligible by title and abstract, full-text articles were obtained. Full-text articles were critically reviewed independently (LEK, SM and FS) to assess eligibility. Studies published prior to 2007 were excluded as most studies focused on the use of tincture of opium which is no longer utilized for the management of NAS. Reasons for exclusions were documented. Any disagreement in study eligibility criteria was resolved through discussion and consensus or by consulting the principle investigator (LEK). Studies were excluded if they did not describe NAS health outcomes or if the full text was not available in a language mastered by our team (English, French, Spanish or Dutch). Only infants with an NAS diagnosis (irrespective of the diagnostic tool utilized) following known opioid exposure in utero were included regardless of concomitant substance exposure.

### Data extraction

Data were extracted independently and in duplicate by two reviewers (SM and FS). Disagreements were resolved by consultation with the principal investigator (LEK). A standardized table was utilized for data extraction which included the following information: year of publication, corresponding author and contact information, study design (randomized controlled tried, cohort study, quality improvement, case series, case report), NAS intervention type (pharmacologic or nonpharmacologic), intervention group, control group, randomization, sample size, study objective in full text, method of NAS diagnosis, frequency of monitoring NAS symptoms, duration of exposure, type of maternal exposure (methadone, suboxone, buprenorphine, illicit opioids, benzodiazepines, cocaine, and so forth), study inclusion criteria, study exclusion criteria, primary outcomes, secondary outcomes, and justification for outcome choice. An outcome was included as reported if it was included in the methods, results or discussion sections. The outcome was placed under “primary outcome” if it was explicitly stated as the primary outcome in the study, it was the only outcome reported, or it was implicit in their data reporting (i.e., used in the sample size calculation). Composite outcome measures were separated to gauge the full breadth of definitions utilized in primary research studies.

### Categorizing similar outcomes

Considerable heterogeneity in the terminology used to report outcomes was noted during the data extraction. Outcomes of similar themes were grouped during the data analysis process. For instance, “days of infant opioid treatment”, “length of infant methadone therapy” and “duration of infant oral morphine therapy” were all included in the outcome category “duration of pharmacotherapy for NAS”.

### Assignment of outcome category to core areas

Outcomes reported as either primary and/or secondary were assigned to one of the four core areas plus adverse events as defined in OMERACT Filter 2.0 [27]. OMERACT is a conceptual framework to ensure that a comprehensive set of outcomes is selected to formulate a core outcome set (COS). Its core areas encompass content that is measurable in a trial that include both patient-centered and intervention-specific information. These four core areas include “death”, “life impact”, “resource use” and “pathophysiologic manifestations”. OMERACT recommends that “adverse events” should be measured within the core areas [27].

## Results

**Fig. 1:**
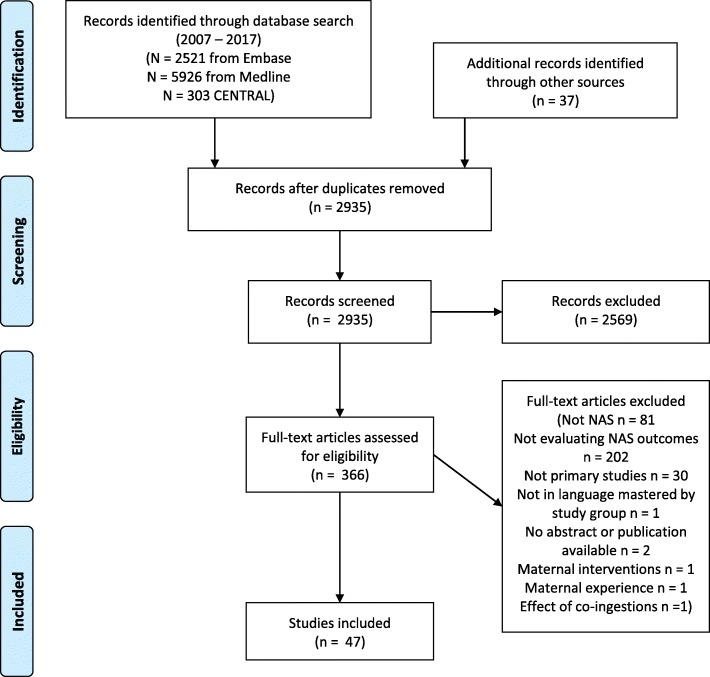
Prisma article selection flow diagram. NAS neonatal abstinence syndrome

The search strategy (Additional file 1) identified 2935 unique articles for screening (Fig. 1); a total of 47 original research publications met the specified inclusion criteria and were included in this review. Reference lists for three recently published NAS reviews and systematic reviews were evaluated and no additional relevant articles were identified [1, 3, 28]. The characteristics of the included studies are outlined in Table 1. The 47 articles published outcomes from ten randomized controlled trials, 21 retrospective cohort studies, five prospective cohort studies, one qualitative analysis, three case series, and one case report. The remaining studies utilized quality improvement methodologies (four studies), combined retrospective and prospective cohort analysis (one study), and a prospective within-subject analysis (one study). There were no disagreements in the study inclusion process that could not be resolved through discussion.

**Table 1:** Characteristics of included studies

First author (ref.)	Year	Intervention type	Intervention	Study type	Region of origin	Sample Size
Ebner [29]	2007	P	Morphine	PCS	Austria	53
Abrahams [30]	2007	NP	Rooming-in	RCS	Canada	106
Mazurier [31]	2008	P	Morphine	CS	France	37
Colombini [32]	2008	P	Morphine	PCS	France	22
Jansson [33]	2008	NP	Breast feeding	PCS	USA	16
Kraft [34]	2008	P	Sublingual buprenorphine	RCT	USA	26
Agthe [35]	2009	P	Clonidine	RCT	USA	80
Leikin [36]	2009	P	Clonidine	CS	USA	14
Abrahams [37]	2010	NP	Rooming-in	RCS	Canada	952
Saiki [38]	2010	NP	Postnatal ward	RCS	UK	60
Esmaeili [39]	2010	P	Clonidine and chloral hydrate	RCS	Germany	133
O’Mara [40]	2010	P	Clonidine	CR	USA	1
Isemann [41]	2011	NP	Breastfeeding	RCS	USA	128
Kraft [42]	2011	P	Sublingual buprenorphine	RCT	USA	24
McQueen [43]	2011	NP	Breastfeeding	RCS	Canada	28
Schwartz [44]	2011	NP	Auricular acupressure	RCT	USA	76
Backes [45]	2012	NP	Outpatient weaning	RCS	USA	121
Filippelli [46]	2012	NP	Noninsertive acupuncture	CS	USA	54
Murphy-Oikonen [47]	2012	NP	Clinical practice guideline	RCS	Canada	90
O’Connor [48]	2013	NP	Breastfeeding	RCS	USA	85
Welle-Strand [49]	2013	NP	Breastfeeding	Mixed method (RCS and PCS)	Norway	124
Surran [50]	2013	P	Clonidine adjunct	RCT	USA	68
Hall [51]	2014	P	Weaning protocols for morphine, methadone, buprenorphine	RCS	USA	547
Bhatt-Mehta [52]	2014	P	Methadone	RCS	USA	60
Smirk [53]	2014	NP	Home-based detoxification	RCS	Australia	118
Asti [54]	2015	NP	Implementation of standardized protocol	QI	USA	92
Hall [55]	2015	P	Methadone weaning protocol	PCS	USA	360
Liu [56]	2015	NP	Breastfeeding	RCS	Australia	194
Hall [57]	2015	P	Standardized weaning protocol	RCS	USA	981
Raith [58]	2015	NP	Laser acupuncture	RCT	Austria	28
Brown [59]	2015	P	Methadone	RCT	USA	31
Bada [60]	2015	P	Morphine	RCT	USA	31
Lee [61]	2015	NP	Combined in/outpatient	RCS	USA	139
Nayeri [62]	2015	P	Phenobarbital	RCT	Iran	60
Young [63]	2015	P	Oral morphine	RCS	USA	26
Newman [64]	2015	NP	Rooming-in	PCS	Canada	45
Kelly [65]	2015	NP	Home weaning	RCS	Canada	80
Hall [66]	2016	P	Buprenorphine	RCS	USA	201
Ibach [67]	2016	P	Methadone	RCS	USA	50
Hahn [68]	2016	NP	Massage	Qualitative thematic analysis	USA	8
Holmes [69]	2016	NP	Rooming-in	QI	USA	163
Short [70]	2016	NP	Breastfeeding	RCS	USA	3725
Patrick [71]	2016	NP	Evidence-based practice guidelines	QI	USA	3458
Kraft [72]	2017	P	Buprenorphine	RCT	USA	63
Zuzarte [73]	2017	NP	Vibrotactile stimulation	Prospective within-subject study	USA	26
Grossman [74]	2017	NP	Standardization and inpatient unit	QI	USA	287
Howard [75]	2017	NP	Parental presence	RCS	USA	86

*CR* case report, *CS* case series, *NP* non-pharmacologic, *P* Pharmacologic, *PCS* prospective cohort study, *QI* quality improvement, *RCS* retrospective cohort study, *RCT* randomized controlled trial

The characteristics of the included publications are displayed in Table 1. The country of origin for the studies included the USA (*n* = 31), Canada, (*n* = 6), France (*n* = 2), Australia (*n* = 2), Austria (*n* = 2), Norway (*n* = 1), the UK (*n* = 1), Iran (*n* = 1) and Germany (*n* = 1). Of the included studies, 21 evaluated pharmacologic regimens while 26 assessed nonpharmacologic interventions for NAS. The median sample size was 78 neonates (range 1 to 345).

### Description of outcomes reported in primary research studies

**Fig. 2:**
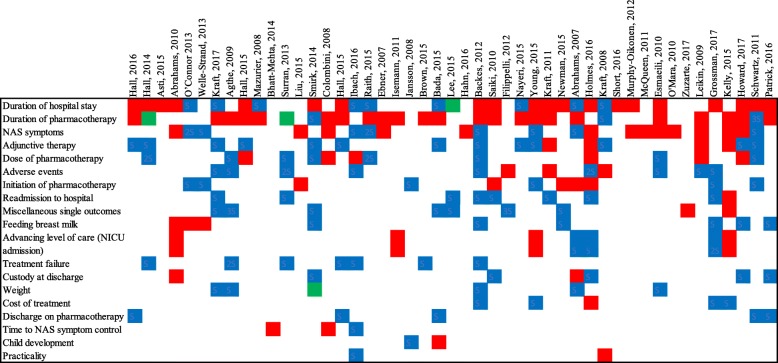
Outcome matrix of 234 outcome terms (rows) for 47 primary research studies (columns). Red signifies a primary outcome. Blue signifies a secondary outcome. Green signifies a primary and secondary outcome reported in the same article that fit within the same outcome category. NAS neonatal abstinence syndrome, NICU neonatal intensive care unit

A total of 107 primary outcomes and 127 secondary outcomes were reported in the included 47 studies. Of these outcomes, 67 primary and 94 secondary outcomes, respectively, were unique ‘terms’ that were not used in any other study. The individual outcomes were mapped into outcome categories as illustrated in Table 2. For ambiguous reported outcomes, the full text of the article was reviewed to determine how it was measured to classify the outcome into an outcome category. For instance, “effectiveness of methadone for the treatment of NAS” was the primary outcome in one article [52]. The surrogate markers of “effectiveness of methadone for the treatment of NAS” were “time since need for pharmacological treatment was established”, “methadone dose in mg/kg/day”, “methadone dosing interval” and “Lipsitz scores”. These surrogate markers were separated as individual outcomes to accurately reflect specific outcome measures. Fifteen outcomes were mapped into the outcome category of “miscellaneous single outcomes”. The most frequently reported outcome category was “duration of hospital stay”, which was reported in 30 primary research studies (63.8%) as a primary or secondary outcome. Twenty-one of these primary research studies reported “duration of hospital stay” as a primary outcome measure. As noted in Table 2, there is heterogeneity in how the most commonly reported outcome (duration of hospital stay) is measured in research studies using six different definitions. Figure 2 demonstrates the distribution of primary and secondary outcome terms across individual research studies. There is also a wide range in the number of outcomes reported (1 to 12) per study. For instance, only seven (15%) of studies reported all three of the most frequently reported outcome categories (duration of hospital stay, duration of pharmacotherapy, and presence of NAS symptoms). Heterogeneity of outcome selection is reflected by the large number of primary (53/107) and secondary (76/127) outcomes that were only reported in a single study. The timing of outcome measurement was poorly reported across studies and was inconsistent. For example, neurobehavior was measured in two studies: one study reported Bayley Scale scores at 1 year and the second reported the NICU Network Neurobehavioral Scale (NNNS) at 5–7 days old and at 44 weeks postmenstrual age. Withdrawal severity scoring outcomes were reported after 1 week of treatment, six times daily, and “according to hospital policies”. Training of outcome assessors was only mentioned for seven primary outcomes and six secondary outcomes. Training protocols were only provided in one study. Outcome assessors were described for 12 primary and 14 secondary outcomes, and were mostly clinicians (physicians and/or nurses, *n* = 9/12 and *n* = 12/14, respectively). The study design may affect the outcomes reported as demonstrated in Table 3. For the most commonly utilized study designs such as retrospective cohort studies (*n* = 21), randomized controlled trials (*n* = 10) and prospective cohort studies (*n* = 5) there was less variability in outcomes selected compared to quality improvement studies and case reports. Cohort studies and clinical trials reported “duration of hospital stay” and “duration of pharmacotherapy” most frequently. Qualitative improvement studies also reported length of stay, which reflect the nature of the study design, targeted to reduce adverse events and resource consumption. Case series/reports, classically written to report unusual or novel occurrences, tended to report “miscellaneous single outcomes” that were not studied at a larger scale in cohort or randomized controlled trials.

**Table 2:** Variation in definition of outcomes reported

Outcome	Reported outcome definition
Duration of pharmacotherapy (*n* = 32)	Days of opioid treatment
	Duration of treatment for NAS symptoms
	Duration of pharmacotherapy for NAS/NOWS
	Days of treatment
	Duration of treatment required for NAS resolution
	Number of days on morphine
	Duration of opioid treatment
	Duration of oral morphine treatment
	Length of opioid treatment
	Length of methadone therapy
	Duration of phenobarbital treatment
	Total phenobarbital treatment days
	Total treatment duration (hospital + home)
	Length of treatment
	Days on DTO
	Days on any form of pharmacologic support
Duration of hospital stay (*n* = 31)	Days of inpatient hospitalization
	Neonatal length of stay
	Length of inpatient stay
	Duration of hospital stay
	Mean infant hospital stay
	Average length of stay between hospitals
NICU level of care (*n* = 8)	NICU admission
	Length of NICU stay
	Admission to level II nursery or chronic care unit
	Adverse event—unplanned ICU transfer
	Transferred to NICU from inpatient unit
Breastfeeding or human milk nutrition (*n* = 12)	Breastfeeding during hospital stay
	Breastfeeding at discharge
	Initiation of breastfeeding
	Breastfeeding rates 6–8 weeks of age
	Rate of breastfeeding
	Duration of breast feeding
	Breastfeeding
	Taking ≥50% of feed as breast milk at discharge
	Discharge on human milk
Opioid withdrawal symptoms (*n* = 24)	Neonatal substance withdrawal
	Withdrawal symptoms
	Time of onset of symptoms
	Severity of NAS/NOWS
	Average daily score
	Mean NAS score
	Intensity of withdrawal
	Control of withdrawal symptoms
	Number of times NAS score >8
	Mean Finnegan withdrawal scores
	Treatment effectiveness
	Duration of neonatal abstinence syndrome
	Severity of NAS (mean peak NAS)
	NAS scores (undefined time point)
	Highest Finnegan score
	Time to highest Finnegan score
	Highest NAS score
	Prevalence of symptoms of NAS
Custody at discharge (*n* = 7)	Custody status at discharge
	Discharge of babies in the custody of their mothers
	Child placed in foster care
	Discharge in parental care
	Discharge from hospital with their biological family
Dose of pharmacotherapy (*n* = 18)	Total dose (mg/kg) of methadone administered
	Range of morphine doses (mg/kg/day)
	Morphine doses during the first 38 days of treatment
	Maximum morphine dose (mean mg/kg/day)
	Initial median opioid dose (methadone or morphine PO)
	Cumulative morphine dose
	Total opioid dose
	Maximum clonidine dose
	Highest mean methadone dose given
	Amount of DTO required to treat NAS
	Mean total morphine dose
	Maximum amount of morphine (mL/kg)
	Time to maximum amount of oral morphine
	Median dosages over time
	Average phenobarbital dose
Addition of adjunctive therapy (*n* = 17)	Phenobarbital adjunct therapy
	Percentage treated with an adjuvant medication
	Adjunct medications
	Addition of a second agent (clonidine or phenobarbital)
	Amount of pharmacologic support
	Addition of a second agent
Initiation of pharmacotherapy (*n* = 11)	Treated with morphine
	Pharmacologic treatment for NAS
	Need for treatment of NAS
	Need for pharmacotherapy with morphine
	Percentage of cohort requiring oral morphine
Weight (*n* = 7)	Weight gain
	Discharge weight
	Weight loss greater than 10% in the first week of life
	Time to regain birthweight
	Poor weight gain
Time to symptom control (*n* = 3)	Duration of treatment to achieve Lipsitz score below 4
	Treatment response
	Time to symptom control after initiation of therapy
Cost of treatment (*n* = 6)	Costs for all opioid-exposed infants
	Costs for all opioid-exposed infants treated pharmacologically
	Average hospital cost per infant
	Total cost of treatment
	Average total cost of hospitalization (direct and indirect)
	Cost-effectiveness
Development (*n* = 2)	Infant neurobehavior
	Neurobehavior
Adverse events (*n* = 14)	Safety of noninsertive acupuncture
	Neonatal safety (adverse drug reactions)
	Safe
	Safety (adverse events)
	Seizures
	Mortality in hospital
	Infant death
	Unintended side effects
Readmission to hospital (*n* = 9)	Return to hospital for withdrawal treatment
	Readmission to hospital
	Readmission in 1 week postdischarge
	Readmission in the first year of life
	Hospital readmission 2 months following discharge
	30-day readmissions (all cause)
	30-day readmission (withdrawal)

*n* represents the number of times an outcome category was reported as either a primary or secondary outcome; difference between number of times an outcome category was reported and the number of articles reporting an outcome category is secondary to the use of composite outcomes

*DTO* diluted tincture of opium, *ICU* intensive care unit, *NAS* neonatal abstinence syndrome, *NICU* neonatal intensive care unit, *NOWS* neonatal opioid withdrawal syndrome, *PO* per oral, *mL*mililiters, *kg* kilograms

**Table 3:** Study design and top three most commonly reported outcome categories

Type of study	First most commonly reported outcome category	Second most commonly reported outcome category(ies)	Third most commonly reported outcome category(ies)
Retrospective cohort study (*n* = 21)	Duration of hospital stay (16%)	NAS symptoms (13%)	Duration of pharmacotherapy (12%)
Randomized controlled trial (*n* = 10)	Duration of pharmacotherapy (22%)	Duration of hospital stay (12%)	Adjunctive therapy (10%)
			Adverse events (10%)
Prospective cohort study (*n* = 5)	Dose of pharmacotherapy (22%)	Duration of pharmacotherapy (17%)	Duration of hospital stay (11%)
			NAS symptoms (11%)
			Initiation of pharmacotherapy (11%)
Quality improvement (*n* = 4)	Duration of hospital stay (15%)	Adverse events (12%)	Initiation of pharmacotherapy (8%)
		Cost of treatment (12%)	Readmission to hospital (8%)
		Advancing level of care (NICU admission) (12%)	Feeding breast milk (8%)
			Custody at discharge (8%)
Case series and case report (*n* = 4)	Miscellaneous single outcomes (25%)	Duration of pharmacotherapy (17%)	Duration of hospital stay (8%)
		NAS symptoms (17%)	Dose of pharmacotherapy (8%)
		Adverse events (17%)	Adjunctive therapy (8%)
Qualitative study (*n* = 1)	NAS symptoms (100%)		
Mixed method (*n* = 1)	Feeding breast milk (100%)	NAS symptoms (25%)	
		Initiation of pharmacotherapy (25%)	
Prospective within-subject study(*n* = 1)	Miscellaneous single outcomes (100%)		

*NAS* neonatal abstinence syndrome

### Assignment of primary outcomes to OMERACT core areas

**Fig. 3:**
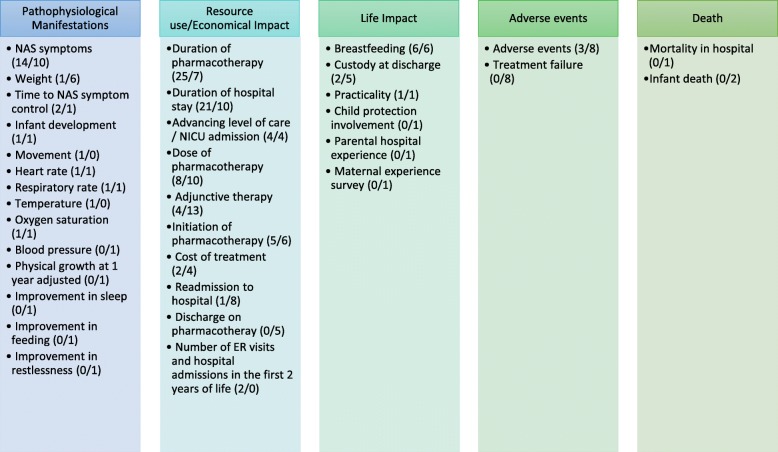
Assignment of outcome terms to OMERACT 2.0 core areas. Parentheses show the number of studies in which the outcome was used as primary outcome/used as secondary outcome. See Table 2 for definitions of adverse events and treatment failure. ER emergency room, NAS neonatal abstinence syndrome, NICU neonatal intensive care unit

One hundred and seven primary outcomes and 127 secondary outcomes were mapped to core domains defined by OMERACT filter 2.0 in Figure 3 [27]. The core area most commonly studied was “Resource Use/Economical Impact” through 72 primary outcomes and 67 secondary outcomes. This was followed by “pathophysiological manifestations” covered by 23 primary outcomes and 26 secondary outcomes and “life impact” examined by nine primary outcomes and 15 secondary outcomes. “Adverse events” were reported as three primary outcomes and 16 secondary outcomes. “Death” was not reported as a primary outcome in any study and was reported as three secondary outcomes.

## Discussion

This review summarizes the primary and secondary outcomes that are reported in current primary research studies (randomized controlled trials, cohort studies, qualitative analysis, case series, case reports, quality improvement studies) of neonates with NAS. We found substantial heterogeneity in outcomes selected and poor overall standardization of definitions and timing of measurement across commonly reported outcomes. These findings have important implications for clinical practice and for research as inconsistency in outcome selection, definition and measurement yields results that cannot be combined in meta-analyses. For example, “weight” is used as a proxy for NAS-related feeding concerns in seven studies, but it was defined as “weight gain”, “discharge weight”, “weight loss greater than 10% in the first week of life”, “poor weight gain” and “time to regain birthweight”, jeopardizing comparison and synthesis of study results. This finding further substantiates the challenge in data synthesis of quantitative data if a range of time points are utilized to measure one outcome.

In our systematic review, 47 original research articles reported 107 primary outcome measures resulting from the use of composite outcomes. These findings are consistent with a recent overview of Cochrane systematic reviews in neonatology which found that over half of recent reviews were inconclusive secondary to heterogeneity of the literature and poor methodologic quality of the studies [76]. Poor outcome definitions may contribute to reporting bias. Chan et al. noted that 62% of trials had primary outcomes changed, introduced or omitted in the process of protocol to publication [77]. Inconsistently selected and defined outcomes led to results that are not reproducible, transparent or comparable [78]. Timing of outcome measure was poorly reported across studies, which may be understandable for outcomes which measure time (for example, duration of time in hospital or duration of treatment) but create challenges for interpreting time-dependent variables such as neurodevelopment and withdrawal severity scoring. Training and descriptions of outcome assessors were poor and, given the subjective nature of some withdrawal scoring criteria, this creates barriers for interpretation and replication. Not only does this contribute to research waste, but also can create misleading conclusions that clinicians use to inform patient care.

COSs have been proposed as a method of standardizing outcome selection, measurement and reporting to optimize research data in terms of transparency, reproducibility and clinical utility [27, 79, 80]. The absence of a COS results in: 1) meaningful outcomes (i.e., patient preferences) being overlooked or lost in study design; 2) inconsistent definitions or measurement tools used across similar studies; and 3) choosing outcomes for publication based on the results of individual studies (reporting bias) [27, 79, 80]. Thus, establishment of a COS representing a minimum set of outcomes that must be measured and reported in all research on NAS interventions and prognosis is critical to improving research quality and evidence-informed care. This review is the first step in a series of methods to develop consensus on what should be measured in all studies on neonatal withdrawal. Consistency in reporting would enhance the value of NAS/NOWS literature by reducing reporting bias of prespecified outcomes and ensuring that research efforts contribute clinically relevant information. In addition, standardization of outcomes would allow trial results to be compared, contrasted and combined in systematic reviews and meta-analyses to create a more robust foundation of knowledge for clinical decision-making and funding allocation [79].

Furthermore, this study found that outcomes related to the core area “life impact” are scarce in existing NAS literature, suggesting that public stakeholder involvement in outcome selection is limited to date. With the rise of substance use disorders comes an increase in neonates with in utero exposure who are experiencing NAS/NOWS. Key stakeholders, the parents and caregivers, are often in a situation of insufficient community support due to lack of knowledge and awareness about the life impact of NAS for that infant and family. Most outcomes reported are related to “resource utilization” reflecting the increasingly high economic burden of NAS, the main concern from the policy-maker point of view. While important, these outcomes do not provide evidence for clinicians on the optimal care for the infant, the mother and/or dyad involved. By estimating the burden of NAS based on nearsighted benchmarks such as “length of hospital stay” or “duration of pharmacotherapy”, while simplifying the methodology of studies, the cost to society may be grossly underestimated. There are still principle variables such as the impact of in utero opioid exposures on physical health, neurodevelopmental, psychiatric health and psychosocial interactions of the growing child that have yet to be explored. How do these long-term sequelae for the child affect parents, families and support workers?

### Strengths and limitations

The strengths of our study are the inclusion of all primary research studies, following a preregistered protocol, and methods following the PRISMA guidelines [81]. A comprehensive search of the bibliographies for the studies included and recent systematic reviews was undertaken to identify any articles missed in our search strategy. Furthermore, the steering committee reviewed the list of included articles and were invited to suggest articles that were not identified using our search strategy. The main limitations to our review are the time boundaries of 2007–2017 for the search strategy and most included studies reported data from western countries which may limit external validity. This is, however, a limitation of the literature available and we do not feel this is related to our search or selection process. In this review, we did not contact the research groups for missing information. The aim of our study was to abstract primary and secondary outcomes and, as such, we did not intend to assess the rigor to which the studies reported this information as missing data are unlikely to affect the conclusions formulated here.

The conclusions from this systematic review advocate for the formation of a COS for NAS/NOWS to establish consistently reported outcomes with standardized definitions. Other COSs and studies have demonstrated that public involvement leads to research that is comprehensive and relevant to the patients receiving evidence-based interventions [82]. Since 2010, the COMET initiative has set out to establish standardized sets of outcomes for intervention clinical trials and clinical audits [83]. Their overarching objective is to limit outcome heterogeneity, include relevant outcomes, and reduce outcome reporting bias through the development of COSs. Trials evaluating the effectiveness of a treatment should measure and report at least each outcome in the COS. However, the primary outcome should be chosen and powered to answer the research question. COS development should be comprehensive and include outcomes relevant for multiple stakeholders from patients/families, clinicians and scientists.

Recent expert reviews in the *Journal of the American Medical Association* [28] and the *New England Journal of Medicine* [3] concluded several areas of uncertainty including standardized and validated assessment tools, optimal medication treatment regimen, optimal location for weaning, long-term neurodevelopment and family outcomes in NAS/NOWS that require further research. Given the heterogeneity in outcome selection, measurement and reporting highlighted by this study, it is imperative and timely to devise a COS for NAS/NOWS. A NAS/NOWS COS will standardize research methodology to reduce research waste and enhance transparency for clinical decision-making. With a scarcity of knowledge on the long-term patient and family impact of NAS/NOWS, the COS should reflect the needs and concerns for the stakeholders that are most affected by this condition. A consensus and evidence-based NAS/NOWS COS is underway led by a multidisciplinary international steering committee. The development of this COS includes family interviews to ensure that future studies will evaluate outcome measures that are meaningful to the population it affects the most.

## Conclusion

Inconsistent selection and definition of primary and secondary outcomes exists in the literature of pharmacologic and nonpharmacologic interventions for managing opioid withdrawal in neonates. No studies involved parents in the process of outcome selection. These findings hinder evidence synthesis to generate clinically meaningful practice guidelines. The development of a specific COS reflecting the needs of stakeholders, including families, is required to improve the quality of clinical practice guidelines.

## Supplementary information

**Additional file 1.** MEDLINE search strategy.

## Data Availability

Data and materials are available from the corresponding author on request.

## References

[1] Kocherlakota P. (2014). Neonatal Abstinence Syndrome. PEDIATRICS.

[2] Hudak ML, Tan RC, Committee on Drugs, Committee on Fetus and Newborn, American Academy of Pediatrics (2012). Neonatal drug withdrawal. Pediatrics.

[3] McQueen K, Murphy-Oikonen J (2016). Neonatal abstinence syndrome. N Engl J Med.

[4] Canadian Paediatric Society. Managing infants born to mothers who have used opioids during pregnancy. Available at: https://www.cps.ca/en/documents/position/opioids-during-pregnancy. Accessed 8 July 2018. 10.1093/pch/pxx199 PMC5951077 29769809

[5] Patrick SW, Davis MM, Lehmann CU, Cooper WO (2015). Increasing incidence and geographic distribution of neonatal abstinence syndrome: United States 2009 to 2012. J Perinatol.

[6] Davies H (2016). Neonatal drug withdrawal syndrome: cross-country comparison using hospital administrative data in England, the USA, Western Australia and Ontario, Canada. Arch Dis Child Fetal Neonatal Ed.

[7] O’Donnell M (2009). Increasing prevalence of neonatal withdrawal syndrome: population study of maternal factors and child protection involvement. Pediatrics.

[8] Filteau J, Coo H, Dow K (2018). Trends in incidence of neonatal abstinence syndrome in Canada and associated healthcare resource utilization. Drug Alcohol Depend.

[9] Bérard A, Zhao J-P, Sheehy O (2017). Antidepressant use during pregnancy and the risk of major congenital malformations in a cohort of depressed pregnant women: an updated analysis of the Quebec Pregnancy Cohort. BMJ Open.

[10] Whiteman VE (2014). Maternal opioid drug use during pregnancy and its impact on perinatal morbidity, mortality, and the costs of medical care in the United States. J Pregnancy.

[11] Forray A, Merry B, Lin H, Ruger JP, Yonkers KA (2015). Perinatal substance use: a prospective evaluation of abstinence and relapse. Drug Alcohol Depend.

[12] Lester W (2019). Symptomology associated with in utero exposures to polysubstance in an Appalachian population. Marshall J Med.

[13] Forray A, Foster D (2015). Substance use in the perinatal period. Curr Psychiatry Rep.

[14] Grisham L (2019). Eat, Sleep, Console approach: a family-centered model for the treatment of neonatal abstinence syndrome. Adv Neonatal Care.

[15] Moore JN (2018). The pharmacokinetics and pharmacodynamics of buprenorphine in neonatal abstinence syndrome. Clin Pharmacol Ther.

[16] Schünemann HJ (2019). GRADE guidelines: 22. The GRADE approach for tests and strategies—from test accuracy to patient-important outcomes and recommendations. J Clin Epidemiol.

[17] Guidelines for the identification and management of substance use and substance use disorders in pregnancy. World Health Organization; 2014. Available at: https://apps.who.int/iris/bitstream/handle/10665/107130/9789241548731_eng.pdf;jsessionid=37BB75E1793034A382C7D5A7AB259D4F?sequence=1. Accessed 27 May 2019. 24783312

[18] Murphy K, Coo H, Warre R, Shah V, Dow K (2017). Variations and similarities in clinical management of neonatal abstinence syndrome: findings of a Canadian survey. Paediatr Child Health.

[19] Bagley SM, Wachman EM, Holland E, Brogly SB (2014). Review of the assessment and management of neonatal abstinence syndrome. Addict Sci Clin Pract.

[20] O’Grady MJ, Hopewell J, White MJ (2009). Management of neonatal abstinence syndrome: a national survey and review of practice. Arch Dis Child Fetal Neonatal Ed.

[21] Corr TE, Hollenbeak CS (2017). The economic burden of neonatal abstinence syndrome in the United States. Addict Abingdon Engl.

[22] Winkelman Tyler N.A., Villapiano Nicole, Kozhimannil Katy B., Davis Matthew M., Patrick Stephen W. (2018). Incidence and Costs of Neonatal Abstinence Syndrome Among Infants With Medicaid: 2004–2014. Pediatrics.

[23] Patrick SW (2012). Neonatal abstinence syndrome and associated health care expenditures: United States, 2000-2009. JAMA.

[24] A core outcome set for neonatal abstinence syndrome: Core Outcome Measures in Effectiveness Trials Initiative (COMET). Available at: http://www.comet-initiative.org/studies/details/808. Accessed 27 May 2019.

[25] Kelly LE (2016). A core outcome set for neonatal abstinence syndrome: study protocol for a systematic review, parent interviews and a Delphi survey. Trials.

[26] Osborn DA, Jeffery HE, Cole MJ. Opiate treatment for opiate withdrawal in newborn infants. Cochrane Database Syst Rev. 2010. 10.1002/14651858.CD002059.pub3. 10.1002/14651858.CD002059.pub3 20927730

[27] Boers M (2014). Developing core outcome measurement sets for clinical trials: OMERACT Filter 2.0. J Clin Epidemiol.

[28] Wachman EM, Schiff DM, Silverstein M (2018). Neonatal abstinence syndrome: advances in diagnosis and treatment. JAMA.

[29] Ebner N (2007). Management of neonatal abstinence syndrome in neonates born to opioid maintained women. Drug Alcohol Depend.

[30] Abrahams RR (2007). Rooming-in compared with standard care for newborns of mothers using methadone or heroin. Can Fam Physician.

[31] Mazurier E (2008). Comparison of chlorpromazine versus morphine hydrochloride for treatment of neonatal abstinence syndrome. Acta Paediatr Oslo Nor.

[32] Colombini N (2008). Hospital morphine preparation for abstinence syndrome in newborns exposed to buprenorphine or methadone. Pharm World Sci PWS.

[33] Jansson LM (2008). Methadone maintenance and breastfeeding in the neonatal period. Pediatrics.

[34] Kraft WK (2008). Sublingual buprenorphine for treatment of neonatal abstinence syndrome: a randomized trial. Pediatrics.

[35] Agthe AG (2009). Clonidine as an adjunct therapy to opioids for neonatal abstinence syndrome: a randomized, controlled trial. Pediatrics.

[36] Leikin JB (2009). Use of clonidine in the prevention and management of neonatal abstinence syndrome. Clin Toxicol Phila Pa.

[37] Abrahams RR (2010). An evaluation of rooming-in among substance-exposed newborns in British Columbia. J Obstet Gynaecol Can.

[38] Saiki T, Lee S, Hannam S, Greenough A (2010). Neonatal abstinence syndrome—postnatal ward versus neonatal unit management. Eur J Pediatr.

[39] Esmaeili A (2010). Treatment of neonatal abstinence syndrome with clonidine and chloral hydrate. Acta Paediatr Oslo Nor.

[40] O’Mara K, Gal P, Davanzo C (2010). Treatment of neonatal withdrawal with clonidine after long-term, high-dose maternal use of tramadol. Ann Pharmacother.

[41] Isemann B, Meinzen-Derr J, Akinbi H (2011). Maternal and neonatal factors impacting response to methadone therapy in infants treated for neonatal abstinence syndrome. J Perinatol Off J Calif Perinat Assoc.

[42] Kraft WK (2011). Revised dose schema of sublingual buprenorphine in the treatment of the neonatal opioid abstinence syndrome. Addict Abingdon Engl.

[43] McQueen KA, Murphy-Oikonen J, Gerlach K, Montelpare W (2011). The impact of infant feeding method on neonatal abstinence scores of methadone-exposed infants. Adv Neonatal Care Off J Natl Assoc Neonatal Nurses.

[44] Schwartz L, Xiao R, Brown ER, Sommers E (2011). Auricular acupressure augmentation of standard medical management of the neonatal narcotic abstinence syndrome. Med Acupunct.

[45] Backes CH (2012). Neonatal abstinence syndrome: transitioning methadone-treated infants from an inpatient to an outpatient setting. J Perinatol Off J Calif Perinat Assoc.

[46] Filippelli AC (2012). Non-insertive acupuncture and neonatal abstinence syndrome: a case series from an inner-city safety net hospital. Glob Adv Health Med.

[47] Murphy-Oikonen J, Montelpare WJ, Bertoldo L, Southon S, Persichino N (2012). The impact of a clinical practice guideline on infants with neonatal abstinence syndrome. Br J Midwifery.

[48] O’Connor AB, Collett A, Alto WA, O’Brien LM (2013). Breastfeeding rates and the relationship between breastfeeding and neonatal abstinence syndrome in women maintained on buprenorphine during pregnancy. J Midwifery Womens Health.

[49] Welle-Strand GK (2013). Breastfeeding reduces the need for withdrawal treatment in opioid-exposed infants. Acta Paediatr Oslo Nor.

[50] Surran B (2013). Efficacy of clonidine versus phenobarbital in reducing neonatal morphine sulfate therapy days for neonatal abstinence syndrome. A prospective randomized clinical trial. J Perinatol Off J Calif Perinat Assoc.

[51] Hall ES (2014). A multicenter cohort study of treatments and hospital outcomes in neonatal abstinence syndrome. Pediatrics.

[52] Bhatt-Mehta V, Ng CM, Schumacher RE (2014). Effectiveness of a clinical pathway with methadone treatment protocol for treatment of neonatal abstinence syndrome following in utero drug exposure to substances of abuse. Pediatr Crit Care Med J Soc Crit Care Med World Fed Pediatr Intensive Crit Care Soc.

[53] Smirk CL, Bowman E, Doyle LW, Kamlin O (2014). Home-based detoxification for neonatal abstinence syndrome reduces length of hospital admission without prolonging treatment. Acta Paediatr Oslo Nor.

[54] Asti L, Magers JS, Keels E, Wispe J, McClead RE (2015). A quality improvement project to reduce length of stay for neonatal abstinence syndrome. Pediatrics.

[55] Hall ES, Meinzen-Derr J, Wexelblatt SL (2015). Cohort analysis of a pharmacokinetic-modeled methadone weaning optimization for neonatal abstinence syndrome. J Pediatr.

[56] Liu A, Juarez J, Nair A, Nanan R. Feeding modalities and the onset of the neonatal abstinence syndrome. Front Pediatr. 2015;3. 10.3389/fped.2015.00014. 10.3389/fped.2015.00014 PMC4341509 25767791

[57] Hall ES (2015). Implementation of a neonatal abstinence syndrome weaning protocol: a multicenter cohort study. Pediatrics.

[58] Raith W (2015). Laser acupuncture for neonatal abstinence syndrome: a randomized controlled trial. Pediatrics.

[59] Brown MS, Hayes MJ, Thornton LM (2015). Methadone versus morphine for treatment of neonatal abstinence syndrome: a prospective randomized clinical trial. J Perinatol Off J Calif Perinat Assoc.

[60] Bada HS (2015). Morphine versus clonidine for neonatal abstinence syndrome. Pediatrics.

[61] Lee J, Hulman S, Musci M, Stang E (2015). Neonatal abstinence syndrome: influence of a combined inpatient/outpatient methadone treatment regimen on the average length of stay of a Medicaid NICU population. Popul Health Manag.

[62] Nayeri F (2015). Phenobarbital versus morphine in the management of neonatal abstinence syndrome, a randomized control trial. BMC Pediatr.

[63] Young ME, Hager SJ, Spurlock D (2015). Retrospective chart review comparing morphine and methadone in neonates treated for neonatal abstinence syndrome. Am J Health-Syst Pharm AJHP Off J Am Soc Health-Syst Pharm.

[64] Newman A (2015). Rooming-in care for infants of opioid-dependent mothers: implementation and evaluation at a tertiary care hospital. Can Fam Physician.

[65] Kelly LE, Knoppert D, Roukema H, Rieder MJ, Koren G (2015). Oral morphine weaning for neonatal abstinence syndrome at home compared with in-hospital: an observational cohort study. Paediatr Drugs.

[66] Hall ES (2016). A cohort comparison of buprenorphine versus methadone treatment for neonatal abstinence syndrome. J Pediatr.

[67] Ibach BW, Johnson PN, Ernst KD, Harrison D, Miller JL (2016). Initial dosing and taper complexity of methadone and morphine for treatment of neonatal abstinence syndrome. J Pharm Technol JPT Off Publ Assoc Pharm Tech.

[68] Hahn J (2016). Neonatal abstinence syndrome: the experience of infant massage. Creat Nurs.

[69] Holmes AV (2016). Rooming-in to treat neonatal abstinence syndrome: improved family-centered care at lower cost. Pediatrics.

[70] Short VL, Gannon M, Abatemarco DJ (2016). The association between breastfeeding and length of hospital stay among infants diagnosed with neonatal abstinence syndrome: a population-based study of in-hospital births. Breastfeed Med Off J Acad Breastfeed Med.

[71] Patrick SW (2016). Improving care for neonatal abstinence syndrome. Pediatrics.

[72] Kraft WK (2017). Buprenorphine for the treatment of the neonatal abstinence syndrome. N Engl J Med.

[73] Zuzarte I (2017). Vibrotactile stimulation: a non-pharmacological intervention for opioid-exposed newborns. PLoS One.

[74] Grossman MR (2017). An initiative to improve the quality of care of infants with neonatal abstinence syndrome. Pediatrics.

[75] Howard MB (2017). Impact of parental presence at infants’ bedside on neonatal abstinence syndrome. Hosp Pediatr.

[76] Willhelm C (2013). Systematic Cochrane reviews in neonatology: a critical appraisal. Pediatr Neonatol.

[77] Chan A-W, Hróbjartsson A, Haahr MT, Gøtzsche PC, Altman DG (2004). Empirical evidence for selective reporting of outcomes in randomized trials: comparison of protocols to published articles. JAMA.

[78] Glasziou P (2014). Reducing waste from incomplete or unusable reports of biomedical research. Lancet Lond Engl.

[79] Williamson P, Altman D, Blazeby J, Clarke M, Gargon E (2012). Driving up the quality and relevance of research through the use of agreed core outcomes. J Health Serv Res Policy.

[80] Sinha IP (2012). Standard 5: selection, measurement, and reporting of outcomes in clinical trials in children. Pediatrics.

[81] Shamseer L (2015). Preferred reporting items for systematic review and meta-analysis protocols (PRISMA-P) 2015: elaboration and explanation. BMJ.

[82] Staley K. Exploring impact: public involvement in NHS, public health and social care research. Eastleigh: INVOLVE; 2009. Available at: https://www.invo.org.uk/wp-content/uploads/2011/11/Involve_Exploring_Impactfinal28.10.09.pdf. Accessed 27 May 2019.

[83] Core Outcome Measures in Effectiveness Trials Initiative (COMET). Available at: http://www.comet-initiative.org/.

