# Supplementary material for: Outcome reporting in neonates experiencing withdrawal following opioid exposure in pregnancy: a systematic review
Source: Trials. 2020 Mar 12;21:262. doi: 10.1186/s13063-020-4183-9 (PMC7069160; doi:10.1186/s13063-020-4183-9)
Supplement: Supplementary file 1 — Additional file 1. MEDLINE search strategy. [file 13063_2020_4183_MOESM1_ESM.docx]

Additional file 1: Medline Search Strategy

Database: Ovid MEDLINE(R) 1946 to Present with Daily Update, Ovid MEDLINE(R) In-Process & Other Non-Indexed Citations <July 22, 2017>

Search Strategy:

--------------------------------------------------------------------------------

1 Neonatal Abstinence Syndrome/ (932)

2 (neonat* adj3 Abstinence).tw,kf. (514)

3 ((withdraw* or abstinence) adj3 (substance* or symptom* or syndrome* or drug* or opioid* or opiate*)).tw,kf. (19016)

4 (passive adj3 addiction*).tw,kf. (15)

5 Substance Withdrawal Syndrome/ (20258)

6 3 or 4 or 5 (32102)

7 (infant* or infancy or newborn* or new-born* or baby* or babies or neonat* or toddler*).mp. (1314214)

8 6 and 7 (1861)

9 1 or 2 or 8 (2319)

10 exp Analgesics, Opioid/ (97559)

11 Opiate Substitution Treatment/ (1474)

12 exp Opioid-Related Disorders/ (20813)

13 ("Alfentanil" or "Alphaprodine" or "Buprenorphine" or "Butorphanol" or "Codeine" or "Dextromoramide" or "Dextropropoxyphene" or "Dihydromorphine" or "Diphenoxylate" or "Enkephalin" or "Ethylketocyclazocine" or "Ethylmorphine" or "Etorphine" or "Fentanyl" or "Heroin" or "Hydrocodone" or "Hydromorphone" or "Levorphanol" or "Meperidine" or "Meptazinol" or "Methadone" or "Methadyl Acetate" or "Morphine" or "Nalbuphine" or "Opiate* " or "Opioid*" or "Opium" or "Oxycodone" or "Oxymorphone" or "Pentazocine" or "Phenazocine" or "Phenoperidine" or "Pirinitramide" or "Promedol" or "Sufentanil" or "Tilidine" or "Tramadol").tw,kf. (152491)

14 10 or 11 or 12 or 13 (175249)

15 Pregnancy/ (741128)

16 Prenatal Exposure Delayed Effects/ (23042)

17 Maternal Exposure/ (6314)

18 (pregnan* or prenat*).tw,kf. (470030)

19 15 or 16 or 17 or 18 (860333)

20 14 and 19 (8124)

21 9 or 20 (9634)

22 21 not (exp animals/ not humans/) (8107)

23 22 not 9 (5926)

***************************
